# The First Selenoanhydride in the Series of Chlorophyll a Derivatives, Its Stability and Photoinduced Cytotoxicity

**DOI:** 10.3390/molecules26237298

**Published:** 2021-12-01

**Authors:** Viktor Pogorilyy, Anna Plyutinskaya, Nikita Suvorov, Ekaterina Diachkova, Yuriy Vasil’ev, Andrei Pankratov, Andrey Mironov, Mikhail Grin

**Affiliations:** 1 Department of Chemistry and Technology of Biologically Active Compounds, Medicinal and Organic Chemistry, Institute of Fine Chemical Technologies, MIREA-Russian Technological University, 86 Vernadsky Avenue, 119571 Moscow, Russia; suvorov.nv@gmail.com (N.S.); mironov@mitht.ru (A.M.); michael_grin@mail.ru (M.G.); 2 P. Hertsen Moscow Oncology Research Institute—Branch of the National Medical Research Radiological Centre of the Ministry of Health of the Russian Federation, 2nd Botkinsky pr., 3, 125284 Moscow, Russia; anna2031@rambler.ru (A.P.); andreimnioi@yandex.ru (A.P.); 3 Department of Oral Surgery of Borovsky Institute of Dentistry, I.M. Sechenov First Moscow State Medical University (Sechenov University), Trubetskaya St. bldg. 8\2, 119435 Moscow, Russia; 4 Department of Fundamental Medical Disciplines, Medical Faculty, Moscow Region State University (MRSU), Str. Radio 10 Build 1, 105005 Moscow, Russia; 5 Department of Operative Surgery and Topographic Anatomy, I.M. Sechenov First Moscow State Medical University (Sechenov University), Trubetskaya St. bldg. 8\2, 119435 Moscow, Russia; y_vasiliev@list.ru; 6 Department of Prosthetic Dentistry, Dental Faculty, Kazan State Medical University of the Ministry of Health of Russia, Str. Butlerova 49, 420012 Kazan, Russia

**Keywords:** naturally occurring chlorins, chlorin p_6_, cyclic thioanhydrides, cyclic selenoanhydrides, photodynamic therapy (PDT), photosensitizers (PS)

## Abstract

In this work, we obtained the first selenium-containing chlorin with a chalcogen atom in exlocycle E. It was shown that the spectral properties were preserved in the target compound and the stability increased at two different pH values, in comparison with the starting purpurin-18. The derivatives have sufficiently high fluorescence and singlet oxygen quantum yields. The photoinduced cytotoxicity of sulfur- and selenium-anhydrides of chlorin p_6_ studied for the first time in vitro on the S37 cell line was found to be two times higher that of purpurin-18 and purpurinimide studied previously. Moreover, the dark cytotoxicity increased four-fold in comparison with the latter compounds. Apparently, the increase in the dark cytotoxicity is due to the interaction of the pigments studied with sulfur- and selenium-containing endogenous intracellular compounds. Intracellular distributions of thioanhydride and selenoanhydride chlorin p_6_ in S37 cells were shown in cytoplasm by diffusion distribution. The intracellular concentration of the sulfur derivative turned out to be higher and, as a consequence, its photoinduced cytotoxicity was higher as well.

## 1. Introduction

The physical and chemical properties of selenium, an element belonging to the chalcogen group, are like those of sulfur, which makes it possible to compare not only the elements themselves but also their compounds [1,2,3]. For example, an analogy may be drawn between cysteine and selenocysteine that are proteinogenic amino acids, with the difference that the sulfur atom in the thiol group of cysteine is replaced by a selenium atom in the selenol group of selenocysteine [4]. The latter is found in more than 25 types of protein molecules in mammal organisms [5,6,7]. Selenium is also a member of the group of glutathione peroxidase (GPx) enzymes that are involved in the reduction of hydrogen peroxide to water [8,9].

Many body disorders, including cancer, atherosclerosis, arthritis, and diseases of the cardiovascular system, are attributed to the lack of selenium [10,11]. Compared to sulfur, selenium exhibits higher antioxidant properties due to its higher nuclear charge, atomic mass, ionic and covalent radii, and a smaller ionization energy [12,13,14].

The main interest in selenium and its compounds stems from their wide use as antitumor agents [15,16,17,18]. It is believed that organic compounds of selenium are generators of superoxide anion O_2^−^_, hydrogen peroxide, and hydroxyl radicals that play the role of mediators of oxidative stress and apoptosis, leading to the death of tumor cells [19]. Tumor cells protect themselves by activating an antioxidant system that involves glutathione (GT), glutathione peroxidase (GPx), glutathione S-transferase (GST), glutaredoxin (Grx), thioredoxin (Trx), superoxide dismutase (SOD), and catalase (CAT) [20,21,22]. The latter are oxidized by selenium compounds, thus making a cell vulnerable to oxidative stress.

Selenophene is the simplest heterocyclic compound containing a selenium atom [23,24]. Several inorganic compounds are used as selenating agents in organic synthesis, including phosphorus pentaselenide, selenium oxides, and hydrogen selenide, which cause the cyclization of linear organic molecules to give selenophenes and their derivatives [24,25,26]. The latter are used as efficient antioxidants and extracting agents for the isolation and separation of metals, while some of them have high biological activity or find use as polymeric materials (tetra-substituted selenophene derivatives) [27,28,29].

Cyclic selenium-containing molecules can affect DNA repair, which allows one to use them as cancer-treatment drugs [15,16,19,25]. Selenium derivatives can suppress the expression of HLA-E in tumor cells, thereby making them susceptible to natural killer cells, which leads to tumor-cell death [30,31].

The successful development of photodynamic therapy (PDT) for cancer is closely related to the development of highly efficient drugs, i.e., photosensitizers (PSs) that lack their own bioactivity but, upon irradiation with light of a certain wavelength, pass into an excited state and react with oxygen to generate active oxygen forms, including singlet oxygen and radical anions [32]. The latter possess a cytotoxic effect but do not damage the surrounding normal tissues due to the limited lifetime and free path. An optimal PS should absorb light in the red or near-infrared spectral range, since, in this case, the light penetrates more deeply into a tissue. PSs should have high quantum yields for singlet oxygen and fluorescence to work as theranostics, and should also be amphiphilic compounds with high tropism toward tumors of various genesis [33,34]. Derivatives of natural chlorins meet all the above requirements, taking into account the fact that the pathways of their metabolism and excretion are well-known [35,36].

Earlier, our research group obtained derivatives of natural chlorins containing various heterocycles, annulated with the main macrocycle, as photosensitizers for photodynamic therapy of cancer [37,38,39,40]. 

The aim of this study was to synthesize a previously unknown chlorin p_6_ selenium anhydride and to study its photoinduced cytotoxicity toward a culture of tumor cells of mouse sarcoma S-37.

## 2. Results

**Scheme 1:**
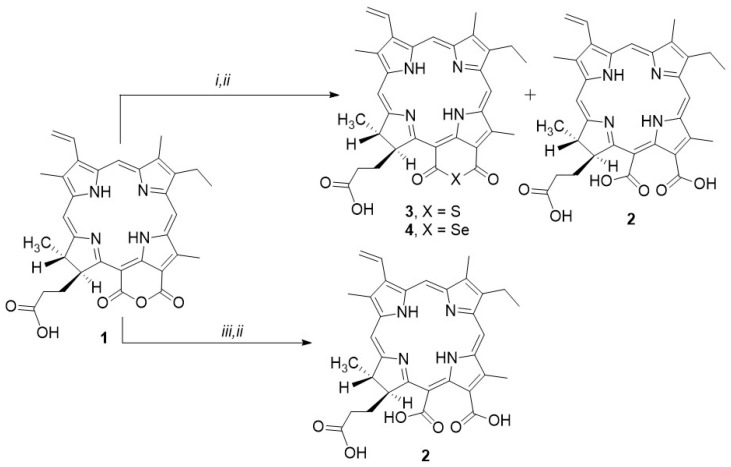
Reagents and conditions: i: 0.5 eq. Na_2_X (X = “S” or “Se”), THF, H_2_O, 25 °C, 240 min, inert argon atmosphere; ii: 4% HCl, 25 °C, 15 min, inert argon atmosphere; iii: 1 eq. Na_2_X (X = “S” or “Se”), THF, H_2_O, 25 °C, 240 min, inert argon atmosphere.

The first cyclic chlorin, p_6_ selenium anhydride (**4**), was obtained by the interaction of purpurin-18 (**1**) and sodium selenide (Scheme 1).

**Scheme 2:**
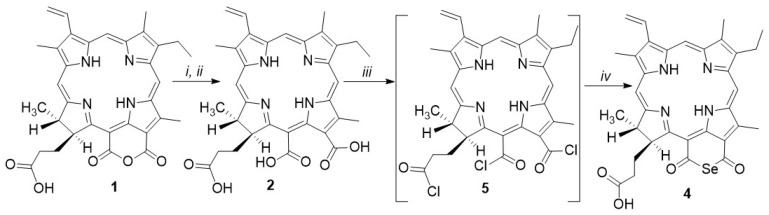
Reagents and conditions: i: NaOH, CH_3_OH, 22 °C, 15 min; ii: 4% HCl, 25 °C, 15 min; iii: SOCl_2_, (Et)_3_N, Et_2_O, 30 min, 22 °C, inert argon atmosphere; iv: Se, LiAlH_4_, THF, 24 h, 22 °C, inert argon atmosphere.

An alternative method for the preparation of cyclic chlorin p_6_ selenium anhydride (**4**) was developed. It was carried out by treatment with a mixture of elemental selenium and lithium aluminum hydride of compound (**5**). Compound (**5**) was obtained from purpurin-18 (**1**) by opening the anhydride ring with a 0.1 M alcoholic solution of potassium hydroxide to form tribasic acid (**2**), which was treated with thionyl chloride to give 13,15,17-trichloroanhydride (**5**) (Scheme 2).

**Figure 1:**
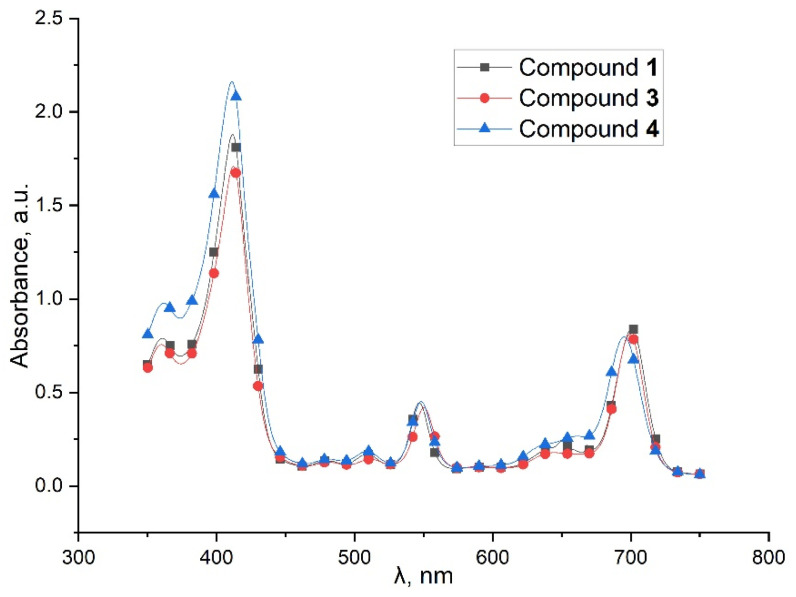
Absorption spectra of compounds (**1**), (**3**) and (**4**). The absorption spectra were obtained at the same solution concentrations, with dichloromethane as a solvent.

The absorption spectrum of selenium anhydride (**4**) is like that of thioanhydride (**3**) and contains a *Q_y_* absorption band that shows a slight hypsochromic shift, with respect to the position in the spectrum of the starting purpurin-18 (**1**). The absorption maxima for compounds (**1**), (**3**), and (**4**) in the long-wavelength region of the spectrum were recorded at 700, 698, and 696 nm, respectively (Figure 1).

**Figure 2:**
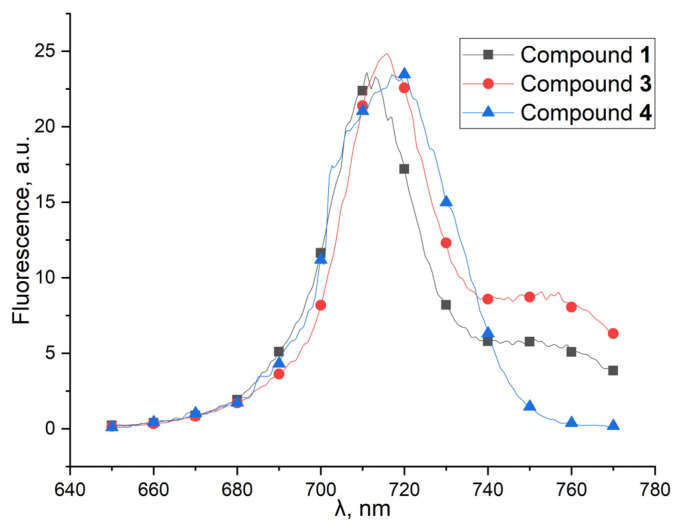
Fluorescence spectra of compounds (**1**), (**3**), and (**4**). The fluorescence spectra were obtained at the same solution concentrations, with dichloromethane as a solvent (excitation wavelength 520 nm).

The fluorescence spectra were recorded with excitation into the absorption band at 550 nm and the maximum fluorescence for compound (**4**) was observed at 722 nm (Figure 2). Chlorin p_6_ thioanhydride (**3**) or chlorin p_6_ selenoanhydride (**4**) have rather high quantum fluorescence yields and amounted to φ = 0.13 and 0.11, respectively. 

The chemical stability of compounds (**1**), (**3**), and (**4**) in 4% aqueous micellar solutions of Kolliphor ELP was investigated over time at different pH values by changing the intensity of the band *Q_y_* in the absorption spectra of the samples.

**Figure 3:**
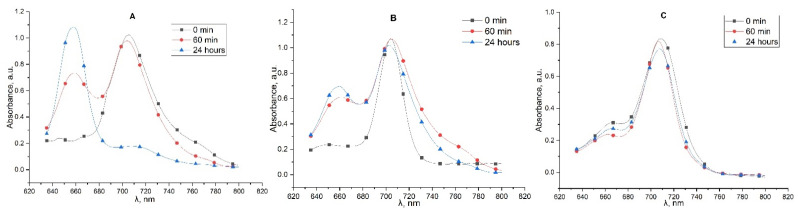
Electronic absorption spectra recorded after various time periods, with 4% micellar solutions of Kolliphor ELP. (**A**) purpurin-18 (**1**); (**B**) chlorin p_6_ thioanhydride (**3**); (**C**) chlorin p_6_ selenoanhydride (**4**).

Figure 3 shows that the stability at pH = 9 increases in the series: purpurin-18 (**1**), chlorin p_6_ thioanhydride (**3**), chlorin p_6_ selenoanhydride (**4**).

In these experiments, we used 4% micellar solutions of Kolliphor ELP solubilizer with purpurin-18 (**1**), chlorin p_6_ thioanhydride (**3**), and chlorin p_6_ selenoanhydride (**4**) with pH = 4. The stability results obtained for compounds (**1**), (**3**), and (**4**) are summarized in Table 1.

The study of photobiological properties included: the photoinduced production of singlet oxygen and ∙OH radicals and the photoinduced activity in vitro.

**Figure 4:**
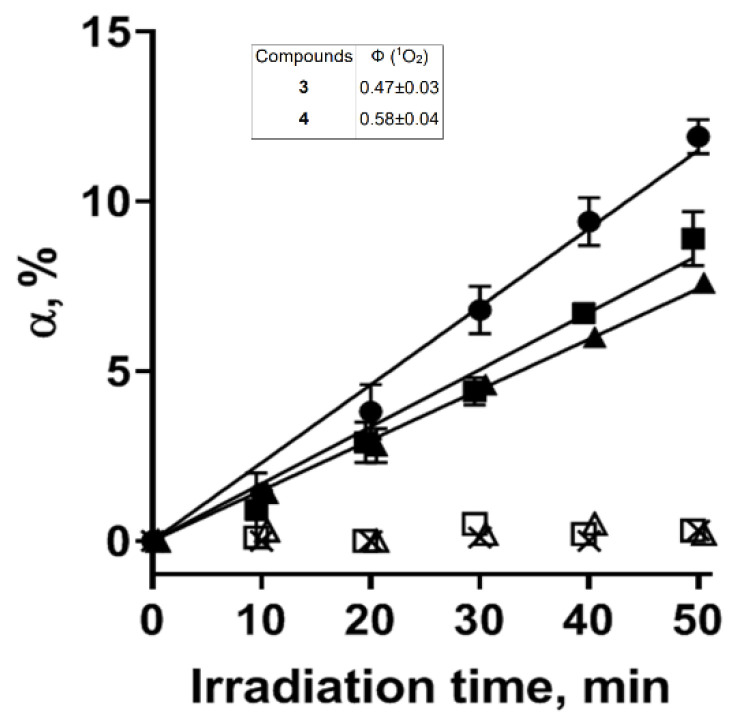
Photoinduced production of singlet oxygen and ∙OH-radicals by chlorin p_6_ thioanhydride (**3**), or chlorin p_6_ selenoanhydride (**4**) in 1% micellar solutions of Kolliphor ELP. The RNO bleaching (α) by ∙OH radicals in the presence of chlorin p_6_ thioanhydride (**3**) or chlorin p_6_ selenoanhydride (**4**) (open triangles and open squares, respectively). The RNO bleaching induced by the reaction of singlet oxygen with histidine in the presence of Rose Bengal (filled circles), chlorin p_6_ thioanhydride (**3**) (triangles), and chlorin p_6_ selenoanhydride (**4**) (filled squares), chlorin p_6_ thioanhydride (**3**), or chlorin p_6_ selenoanhydride (**4**) and sodium azide (crosses). Φ(^1^O_2_) is the quantum yield of singlet oxygen generation.

The photoinduced production of singlet oxygen and ∙OH radicals was investigated using chlorin p_6_ thioanhydride (**3**) or chlorin p_6_ selenoanhydride (**4**) in 1% micellar solutions of Kolliphor ELP (Figure 4).

**Figure 5:**
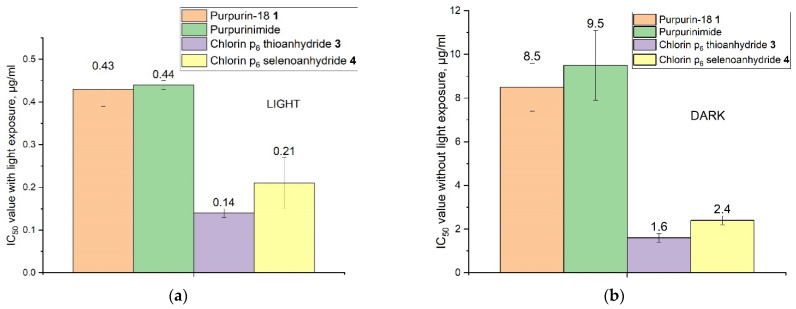
A study of photoinduced activity in vitro of tumor cells of mouse sarcoma S-37. The tested PS was added after 24 h at concentrations from 0.03 μg/mL to 20 μg/mL: (**a**) Photoinduced activity of compounds (**1**), (**3**), (**4**) and purpurinimide, with 4% micellar solutions of Kolliphor ELP. The time of incubation with photosensitizers before exposure to light was 4 h. The power density was 21.0 ± 1.0 mW/cm^2^ and the calculated light dose was 10 J/cm^2^; (**b**) Cytotoxic activity of compounds (**1**), (**3**), (**4**) and purpurinimide, with 4% micellar solutions of Kolliphor ELP.

The photoinduced activity of compounds (**3**) and (**4**) was studied with a culture of tumor cells of mouse sarcoma S-37 (Table S1). The time of incubation with photosensitizers before exposure to light was 4 h, and the incubation time of cells after irradiation was 24 h. The cells were irradiated using a standard light dose of 10 J/cm^2^ and an exposure time of 13 min. The starting compound **1** and purpurinimide were selected as the reference compounds. The tumor cell survival was estimated both visually using an inverted microscope and by the MTT test, whose results are shown in Figure 5.

**Figure 6:**
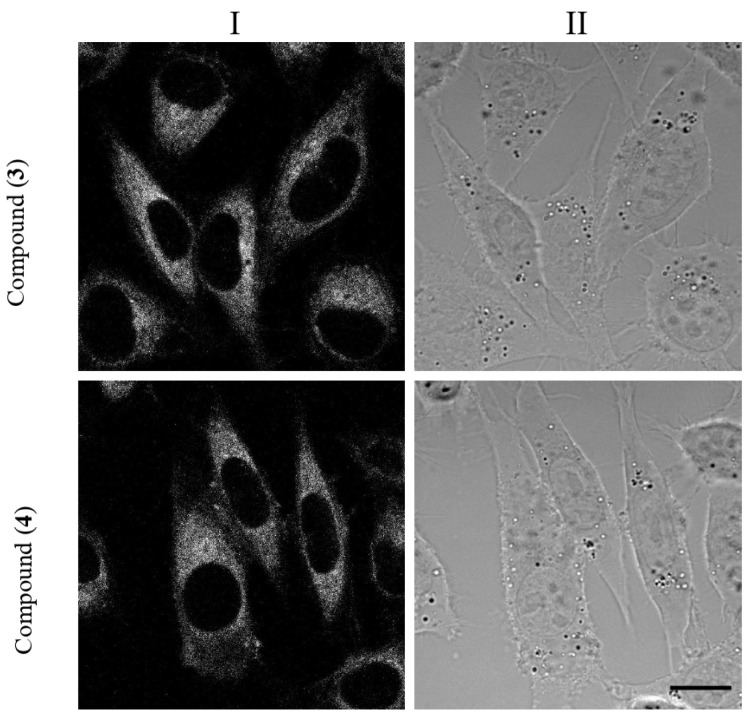
Intracellular distributions of chlorin p_6_ thioanhydride (**3**) and chlorin p_6_ selenoanhydride (**4**) in S37 cells. Column I: confocal fluorescence images describing the subcellular distribution. Column II: transmitted light images of the cells. Bar represents 10 μm. Cells were incubated with chlorin p_6_ thioanhydride (**3**) or chlorin p_6_ selenoanhydride (**4**) (1 μmol L^−1^) for 2 h.

It was found that chlorin p_6_ thioanhydride (**3**) and chlorin p_6_ selenoanhydride (**4**) accumulate efficiently in S37 cells in Figure 6. According to the extraction data, the average cytoplasmic concentrations (ACCs) in S37 cells are 59 ± 9 μmol L^−1^ for chlorin p_6_ thioanhydride (**3**) and 53 ± 8 μmol L^−1^ for chlorin p_6_ thioanhydride (**4**) after incubation with chlorin p_6_ thioanhydride (**3**) or chlorin p_6_ selenoanhydride (**4**) at 1 μmol L^−1^ for 2 h.

## 3. Discussion

Photodynamic therapy (PDT) is an efficient method of cancer treatment where the optimal choice of the photosensitizer (PS) is the major factor in successful tumor elimination [32,33,34]. The PS can be selectively accumulated in a tumor tissue. Some of them possess subcellularly oriented photoinduced cytotoxicity that damages vital cell organelles due to generation of reactive oxygen forms [35,36]. 

One of the promising approaches in the development of new PS involves their chemical design based on molecules of known pigments, namely, by replacing oxygen, nitrogen, and sulfur atoms in the exocycles of purpurin, cycloimide, and thioanhydride with a selenium atom. Since our research team has accumulated extensive experience in the chemical modification of porphyrins and their hydrogenated analogs [37,38,39,40], it seems interesting to create a selenoanhydride exocycle annulated with the main macrocycle to obtain new photosensitizers of chlorin series. As shown previously, a thioanhydride exocycle created in a chlorin molecule results in new physicochemical and spectral properties appearing in the pigment [41,42,43,44,45,46,47]. The improved properties of the sulfur-containing derivatives of porphyrins and chlorins thus obtained made it possible to use them as new complexing agents and potential photosensitizers in photodynamic therapy [48,49]. 

The creation of a six-membered ring annulated with the chlorin macrocycle increases the stability of the pigment molecule and causes a bathochromic shift of the *Q_y_* spectral band. For example, the formation of an imide exocycle results in a bathochromic shift of the main absorption band of purpurinimides to the region around 715 nm and increases the overall stability of the molecule. Moreover, their structure opens rich possibilities for chemical modification. 

The possibility of synthesizing cyclic selenoanhydrides from derivatives of various dicarboxylic acids, including aliphatic, aromatic, and heterocyclic ones, was reported by a few researchers [23,24,25,26]. The synthesis of cyclic chlorin thioanhydride p_6_ (**2**) was first performed by our team [50]. A specific feature of the suggested reaction, which determines the type of the resulting product, is that a particular molar ratio of the initial reagents, i.e., purpurin-18 (**1**) and sodium sulfide, was used. If their ratio was equimolar, no thioanhydride was formed, while the use of a two-fold excess of purpurin-18 (**1**) gave the target thioanhydride.

In this work, the previously identified regularities of this reaction were considered to obtain chlorin p_6_ selenoanhydride (**4**). The reaction of equimolar amounts of purpurin-18 (**1**) and sodium selenide gives only chlorin p_6_ (**2**) in the form of a triacid, whereas the addition of a double amount of purpurin-18 resulted in a mixture of the target selenoanhydride (**4**) and chlorin p_6_ (**2**) (Scheme 1).

**Scheme 3:**
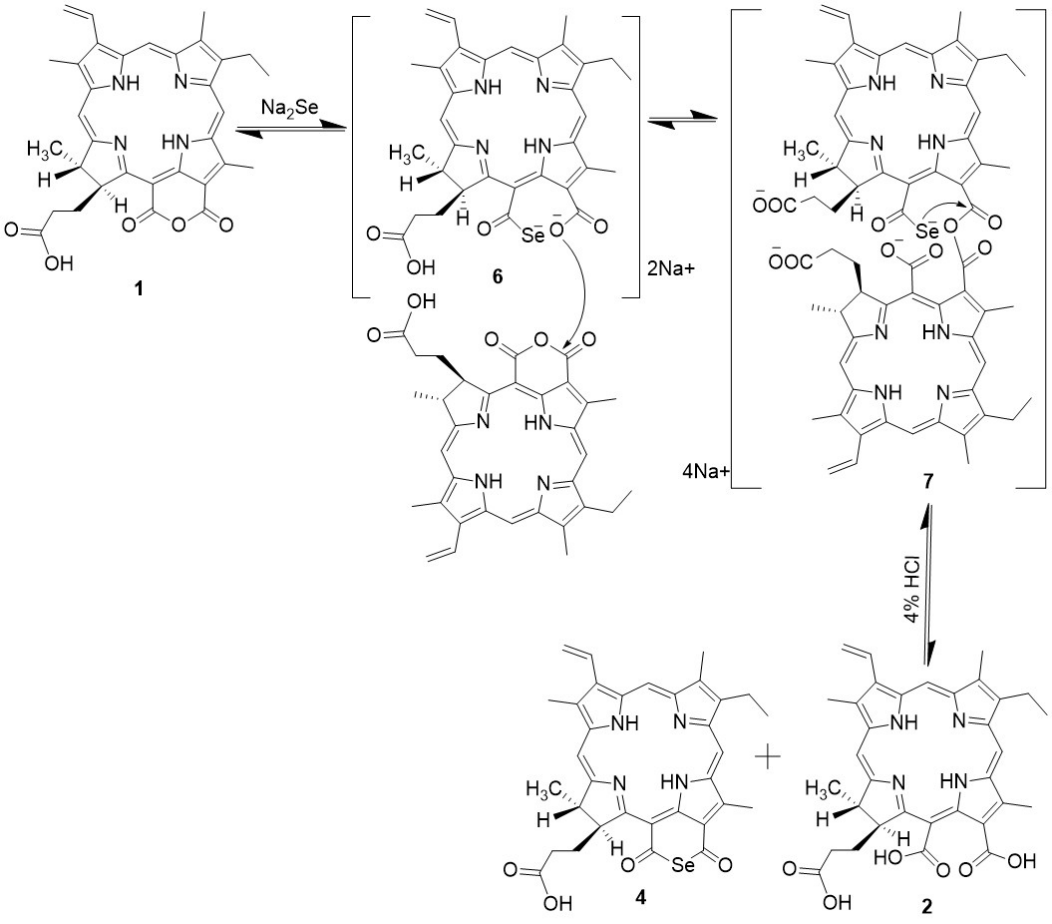
A possible mechanism for the formation of cyclic chlorin p_6_ selenoanhydride.

The previously suggested mechanism of thioanhydride formation can be extrapolated to the formation of the selenoanhydride. Apparently, the initial monoseleniumchlorin p_6_ derivative (**6**) reacts with the second molecule of purpurin-18 (**1**) taken in two-fold excess to give acyclic anhydride (**7**), in which the intramolecular attack of the selenium atom on the adjacent carbonyl carbon atom gives selenoanhydride (**4**) and causes the elimination of a chlorin p_6_ (**2**) molecule (Scheme 3).

The reaction product depends on the molecular ratios of purpurin-18 (**1**) and sodium selenide (Na_2_Se). If a two-fold excess of purpurin-18 is taken (**1**), then acyclic anhydride (**7**) is formed, while at a 1:1 ratio (condition *iii*, Scheme 1), the abovementioned anhydride is not formed, but hydrolysis of monoseleniumchlorin p_6_ derivative (**6**) in an acidic medium leads to the formation of chlorin p_6_ (**2**).

To confirm the structure of compound (**4**), an alternative synthesis was carried out: chlorin p_6_ (**2**) was obtained as a triacid by opening the anhydride ring in purpurin-18 (**1**) with 0.1 M alcoholic solution of potassium hydroxide; tribasic acid (**2**) was converted with oxalyl chloride to 13,15,17-trichloroanhydride (**5**), and, finally, cyclic selenoanhydride (**4**) was obtained by treatment with a mixture of elemental selenium with lithium aluminum hydride (Scheme 2). The LiAlHSeH formed in the reaction is used for the synthesis of diacyl selenides from diacyl chlorides, including the synthesis of selenoanhydrides from various dicarboxylic acid chlorides, both aliphatic and aromatic [51]. The reaction, according to Scheme 2, gave product (**4**).

The target chlorin p_6_ selenoanhydride (**4**) was developed according to Scheme 1 in preparative amounts for further research since it consists of only one-step and it is easy to purify the target compound.

The MALDI-TOF mass spectrum of compound (**4**) contained a signal corresponding to the calculated value for the molecular ion of cyclic anhydride selenium, while the ^1^H NMR spectrum was identical to that of purpurin-18 (**1**). An elemental analysis of the compound (**4**) confirmed the qualitative and quantitative composition of the molecule corresponding to the proposed structure (Figures S1–S3).

In this work, we compared the chemical stability of chlorin p_6_ selenoanhydride (**4**), chlorin p_6_ thioanhydride (**3**), and purpurin-18 (**1**) at different pH values. The experiment involved monitoring the changes in the absorption spectra of the pigments dissolved in 4% aqueous micellar solutions of Kolliphor ELP with time at different pH values. Since the opening of O-, S-, and Se-containing exocycles is accompanied by a significant hypsochromic shift of the long-wavelength absorption band to the 660 nm region, an appearance and intensity increase of the latter would indicate that the structure changes with time (Figure 4).

Figure 4 shows that the stability at pH = 9 increases in the series: purpurin-18 (**1**), chlorin p_6_ thioanhydride (**3**), chlorin p_6_ selenoanhydride (**4**). In 24 h, compound (**1**) is completely converted to chlorin p_6_ in the form of triacid (**2**) and the degree of conversion of chlorin p_6_thioanhydride (**3**) is no higher than 50%, whereas chlorin p_6_ selenoanhydride (**4**) remains stable throughout the experiment.

The choice of a different value, pH = 4, was dictated by the need to simulate the environment of the tumor interstitial tissue, which is known to be acidic [52]. In these experiments, we used 4% micellar solutions of Kolliphor ELP solubilizer with purpurin-18 (**1**), chlorin p_6_ thioanhydride (**3**), and chlorin p_6_ selenoanhydride (**4**) at the same concentration of 1 mmol/L in the buffer solution with pH = 4. The stability results obtained for compounds (**1**), (**3**), and (**4**) are summarized in Table 1. As one can see from Table 1, the stability at pH = 4 increases in the series of compounds: purpurin-18 (**1**), chlorin p_6_thioanhydride (**3**), chlorin p_6_ selenoanhydride (**4**). No complete exocycle opening occurred in the latter, even after 24 h. 

The more electronegative heteroatoms (O, N, S, Se) in the exocycle, the higher the reactivity of the adjacent carbonyl carbon atom in nucleophilic addition/substitution reactions. Apparently, this is the reason for the greater stability of the selenium anhydride ring.

The main indicator of PS efficiency is the singlet oxygen quantum yield, which was determined for two compounds. Chlorin p_6_ thioanhydride (**3**) or chlorin p_6_ selenoanhydride (**4**) have rather high quantum yields of photoinduced singlet oxygen generation and amounted to 0.47 ± 0.03 and 0.58 ± 0.04, respectively. Sodium azide, a well-known specific singlet oxygen quencher, totally suppressed the photoinduced chlorin p_6_ thioanhydride (**3**) or chlorin p_6_ selenoanhydride (**4**)-mediated bleaching of RNO. Chlorin p_6_ thioanhydride (**3**) and chlorin p_6_ selenoanhydride (**4**) do not produce ∙OH radicals under illumination.

The photoinduced activity of compounds (**3**) and (**4**) was studied with a culture of tumor cells of mouse sarcoma S-37 (Table S1). The values presented in Figure 5a show a two-fold increase in photoinduced cytotoxicity in Se-containing derivatives (**4**) compared to the pigments containing an anhydride or imide exocycle. Moreover, the dark cytotoxicity increased four-fold, in comparison with the latter compounds (Figure 5b). Apparently, the increase in the dark cytotoxicity is due to the interaction of the pigments studied with sulfur- and selenium-containing endogenous intracellular compounds. Such interactions can cause a violation of the redox balance in tumor cells, for example, by blocking the glutathione antioxidant system [19] or suppressing DNA repair [15]. The photoinduced cytotoxicity of compounds (**3**) and (**4**) is an order of magnitude higher than the dark cytotoxicity, which is comparable to the previously studied chlorophyll *a* derivatives and commercial PS [53]. 

Intracellular distributions of chlorin p_6_ thioanhydride (**3**) and chlorin p_6_ selenoanhydride (**4**) in S37 cells were shown (Figure 6). In cytoplasm, chlorin p_6_ thioanhydride (**3**) and chlorin p_6_ selenoanhydride (**4**) demonstrate similar diffuse distribution. A consequence of the higher accumulation of compound (**3**) in S37 cells in comparison with compound (**4**) is the higher photoinduced cytotoxicity shown by us, above (Figure 5).

## 4. Materials and Methods

### 4.1. Materials and Instruments

All the solvents used in this work were prepared according to common techniques as described elsewhere. Column chromatography was performed on silica gel 40/60 (Merck, Darmstadt, Germany). Preparative thin-layer chromatography (TLC) was performed on glass plates with silica gel 60 (Merck, Darmstadt, Germany). Analytical TLC was performed on aluminum plates with Silica gel 60 F_254_ with a fluorescent probe (Merck, Darmstadt, Germany).

Absorption and fluorescence spectra were recorded on Shimadzu UV1800 UV/VIS (Shimadzu, Duisburg, Germany) and Shimadzu RF-5301 (Shimadzu, Duisburg, Germany) spectrophotometers in quartz cuvettes (0.4 × 1.0 cm) with an optical path length of 1 cm (spectral slit width 1 nm), with dichloromethane as a solvent. The absorption spectra were recorded in the range of 350–750 nm. Fluorescence spectra were recorded in the range 650–770 nm (excitation wavelength 520 nm), with dichloromethane as a solvent. The fluorescence quantum yield was determined by the relative method. A solution of purpurin-18 in toluene was used as a standard [54]. ^1^H and ^13^C NMR spectra were registered on Bruker DPX 300 (Bruker, Alzenau, Germany). NMR samples were prepared in CDCl_3_. Matrix-assisted laser desorption/ionization (MALDI) mass spectra were recorded on a Bruker Ultraflex TOF/TOF spectrometer (Bruker, Alzenau, Germany), with 2,5-dihydroxybenzoic acid as the matrix. Elemental analysis was performed on EA 1112, Thermo Finnigan, Thermo Scientific, CE Instruments (Thermo Fisher Scientific, Waltham, MA, USA).

Extraction of chlorophyll *a* from *Spirulina platensis*, synthesis of purpurin-18, and synthesis of cyclic thioanhydride of chlorin p_6_ were carried out as described previously [50].

### 4.2. Synthesis

**Cyclic chlorin p_6_ selenoanhydride (4).**

Method 1:

Purpurin-18 (**1**) (177 μmol, 100 mg) was dissolved in tetrahydrofuran (3 mL), and 0.9 M aqueous sodium selenide (1 mL) was added. The reaction mixture was stirred at 25 °C for 240 min in an inert argon atmosphere. The product was extracted with 4% aqueous hydrochloric acid/chloroform (125/25, *v*/*v*) and then purified by column chromatography in dichloromethane/methanol (15/1, *v*/*v*). The yield of the target compound (**4**) was 27 mg (27%).

Method 2:

Chlorin p_6_ (**2**). Compound (**1**) (177 μmol, 100 mg) was dissolved in methanol (5 mL), and 1 M sodium hydroxide (2 mL) was added [50]. The reaction mixture was stirred at 22 °C for 15 min. The product was extracted with 4% aqueous hydrochloric acid/chloroform (50/5, *v*/*v*). The solution was concentrated in a vacuum, and the product was purified by column chromatography (CH_2_Cl_2_/CH_3_OH, 1/1, *v*/*v*). The yield of the target compound **2** was 75 mg (75%).

Thionyl chloride (134 μmol, 26 μL) and triethylamine (10 μmol, 1.5 μL) were added to the solution of compound (**2**) (67 μmol) in diethyl ether (3 mL), and the reaction was performed at 22 °C for 30 min in argon atmosphere. After this, the LiAlHSeH reagent was added to product **7**. The reagent was prepared beforehand by the addition of lithium aluminum hydride (2.6 mg) to a solution of selenium (2.2 mg) in tetrahydrofuran (250 μL). The resulting solution was incubated in an inert argon atmosphere for 24 h at 22 °C, and the compound (**4**) was extracted with chloroform (15 mL) and repeatedly washed with water (150 mL). The solution was concentrated in a vacuum, and the product was purified by preparative TLC (CH_2_Cl_2_/CH_3_OH, 60/1, *v*/*v*). The yield of the target compound (**4**) was 17 mg (42.5%).

UV/VIS (CH_2_Cl_2_) λ max, nm (ε, M^−1^sm^−1^): 412 (9800), 481 (22354), 550 (546), 696 (10506). MALDI-TOF MS *m*/*z* [M+H]^+^ calculated for C_33_H_32_N_4_O_4_Se+H, 627.61 found: 627.67. ^1^H NMR (300 MHz, CDCl_3_, δ, ppm): 9.58 (1H, s, 5H), 9.37 (1H, s, 10H), 8.59 (1H, s, 20H), 7.90 (1H, dd, J = 17.9, 11.4 Hz, 3^1^-H), 6.31 (1H, dd, J = 17.9, 1.3 Hz, 3^2^-H^a^), 6.21 (1H, dd, J = 11.5, 1.3 Hz, 3^2^-H^b^), 5.21 (1H, m, 17-H), 4.39 (1H, q, J = 6 Hz, 18-H), 3.79 (3H, s, 12-CH_3_), 3.63 (2H, m, 8^1^-CH_2_), 3.36 (3H, s, 7-CH_3_), 3.17 (3H, s, 2-CH_3_), 2.76 (1H, m, 17^2^-CH_2_^b^), 2.49 (1H, m, 17^1^-CH_2_^b^), 2.48 (1H, m, 17^1^-CH_2_^a^), 2.02 (1H, m, 17^1^-CH_2_^a^), 1.76 (3H, d, J = 7.3 Hz, 18-CH_3_), 1.67 (3H, t, J = 7.6 Hz, 8^2^-CH_3_), 0.24 (1H, s, NH), and −0.06 (1H, s, NH). ^13^C NMR (75 MHz, CDCl_3_, δ, ppm): 177.40, 176.46, 173.60, 165.21, 164.10, 156.15, 149.94, 145.84, 144.01, 139.22, 137.68, 136.48, 133.68, 131.62, 129.65, 128.28, 123.65, 107.45, 102.99, 94.91,62.67, 54.92, 51.56, 49.14, 32.48, 31.21, 29.63, 23.78, 19.20, 17.30, 12.19, 11.86, and 10.93. Elemental analysis for CHN, calculated for C_33_H_32_N_4_O_4_Se (%): C, 63.15; H, 5.14; N, 8.93; found (%): C, 63.12; H, 5.16; N, 8.91.

### 4.3. A Study of Stability at Different pH Values

The stability was estimated by the variation in the intensity of the *Q_y_* absorption band in the electronic spectra of the samples. 

A total of 4% Micellar solutions of Kolliphor ELP with purpurin-18 (**1**), chlorin thioanhydride p_6_ (**3**), and chlorin p_6_ selenoanhydride (**4**) were added to buffer solutions prepared according to standard methods, so that the final concentration of the pigments in the solution was 1 mmol/L and the pH was 4 or 9. The changes in the absorption spectra of the resulting solutions were monitored for 24 h of incubation.

### 4.4. A Study of Photoinduced Production of Singlet Oxygen and ∙OH-Radicals

The ability of chlorin p_6_ thioanhydride (**3**) and chlorin p_6_ selenoanhydride (**4**) for the photoinduced generation of singlet oxygen was estimated using a 4-nitroso-N, N-dimethylaniline–histidine (RNO) assay [55], as in [56], with minor modifications. Rose Bengal was used as a reference compound with a known quantum yield of singlet oxygen generation (Φ(^1^O_2_) = 0.75) [57]. The RNO-based assay, in the absence of histidine, was used under the same experimental conditions for the detection of ∙OH radicals [58].

### 4.5. A Study of Photoinduced Activity In Vitro

The cells were cultivated under standard conditions at 37 °C in a humidified atmosphere with 5% CO_2,_ in a DMEM environment with the addition of *L*-glutamine (2 mM) and fetal calf serum (10%, PanEco, Moscow, Russia). The photoinduced efficiency was estimated as follows: S37 cells were seeded in a 96-well flat-bottomed plate for microtitration (Costar, New York, NY, USA). The tested PS was added after 24 h at concentrations ranging from 0.03 μg/mL to 20 μg/mL. The time of the incubation with photosensitizers before exposure to light was 4 h. After that, the cells were irradiated with a halogen lamp through a KS-19 broadband filter that passed light with wavelengths above 720 nm, and a water filter 5 cm thick, equipped with a liquid circulation system (λ ≥ 1000 nm). The power density was 21.0 ± 1.0 mW/cm^2^ and the calculated light dose was 10 J/cm^2^. The starting compound **1** and purpurinimide were selected as the reference compounds. After irradiation, the cells were incubated for 24 h under standard conditions. To analyze the PS cytotoxicity and the cytotoxicity (without irradiation), cells were kept for 24 h in the dark. Survival was estimated by visual inspection and colorimetrically using the MTT test. Cell-growth inhibitions of more than 50% were considered biologically significant. This value was calculated as the average of three independent tests.

### 4.6. A Study Intracellular Accumulation

To study intracellular accumulation of chlorin p_6_ thioanhydride (**3**) and chlorin p_6_ selenoanhydride (**4**), cells were seeded on cover glasses in 24-well plates (seeding density of 5 × 10^4^ cells per well) a day before the experiment. Confocal fluorescent images were recorded using a laser scanning confocal microscope Leica TCS SP2 (Leica, Wetzlar, Germany) with a 63× water-immersion lens (numerical aperture 1.2). Lateral and axial resolutions were 0.2 and 1 μm, respectively. Fluorescence of chlorin p_6_ thioanhydride (**3**) and chlorin p_6_ selenoanhydride (**4**) was excited at the 561 nm wavelength and recorded in the 680–750 nm range.

### 4.7. Assessment of Intracellular Accumulation

To estimate the intracellular accumulation of chlorin p_6_ thioanhydride (**3**) and chlorin p_6_ selenoanhydride (**4**) with an extraction technique, 3.5 × 10^6^ cells were incubated with chlorin p_6_ thioanhydride (**3**) or chlorin p_6_ selenoanhydride (**4**) (1 μmol L^−1^) in 5.0 mL of a complete medium for 2 h, washed, detached from a flask, pelleted, and lysed with 0.25% Triton X-100 for 30 min. The concentration of chlorin p_6_ thioanhydride (**3**) and chlorin p_6_ selenoanhydride (**4**) in cellular extract (C_ex_) was measured using fluorescence spectroscopy with reference solutions of chlorin p_6_ thioanhydride (**3**) and chlorin p_6_ selenoanhydride (**4**) of known concentrations in 0.25% Triton X-100. The average cytoplasmic concentration (ACC) of chlorin p_6_ thioanhydride (**3**) and chlorin p_6_ selenoanhydride (**4**) was calculated as

ACC = C_ex_ × V_ex_/(N_c_ × V_cyt_)

where V_ex_, N_c_, and V_cyt_ are an extract volume, a cell number, and an approximate volume of cytoplasm of a cell ((1.2 ± 0.2) × 10^−12^ L), respectively. A total cell volume was assumed to be equal to (1.7 ± 0.2) × 10^−12^ L.

## 5. Conclusions

A selenium-containing derivative of purpurin-18, namely, chlorin p_6_ selenoanhydride, was obtained in this work. The chemical synthesis was performed using two approaches. The first involved a direct reaction of purpurin-18 with sodium selenide, accompanied by a replacement of oxygen in exocycle *E* with a selenium atom. The second involved the reaction of chlorin p_6_ 13,15,17-trichloroanhydride with elemental selenium in the presence of lithium aluminum hydride.

The target compound was characterized by spectral and physicochemical methods. Stability studies showed that the selenoanhydride was more stable under acid (pH = 4) and basic conditions (pH = 9) than the thioanhydride and the starting purpurin-18. The derivatives have sufficiently high fluorescence and singlet oxygen quantum yields. In vitro biological studies of chlorin p_6_ selenoanhydride on the S37 cell line showed a two-fold increase in the photoinduced cytotoxic effect, compared to the previously obtained purpurinimide and the original purpurin-18, while the dark cytotoxicity increased four-fold, in comparison with the latter. We have shown that the chlorin p_6_ thioanhydride accumulates better in S37 cells and has a higher photoinduced cytotoxicity.

## Figures and Tables

**Table 1:** Intensity of the absorption maxima of compounds (**1**), (**3**), and (**4**) under acidic conditions at pH = 4.

Time, min.	Purpurin-18 (1)		Chlorin p_6_ Thioanhydride (3)		Chlorin p_6_ Selenoanhydride (4)	
	Intensity at 700 nm	Content, %	Intensity at 698 nm	Content, %	Intensity at 696 nm	Content, %
0	0.99055	100	0.98381	100	0.99306	100
60	0.72335	73	0.74801	76	0.93902	94.5
1440	0.51956	52.5	0.69036	70	0.89705	90

## Data Availability

The data presented in this study are available from the authors.

## Supplementary Materials

The following are available online. Figure S1: Mass spectrum of compound **4**; Figure S2: ^1^H NMR spectrum of compound **4;** Figure S3: ^13^C NMR spectrum of compound **4**; Table S1: Photoinduced activity of compounds **1**, **3**, **4**, and purpurinimide.

## Sample Availability

Samples of the compounds are available from the authors upon special request and the agreement of the Russian Foundation for Basic Research and the Russian Science Foundation.
