# Supplementary material for: The First Selenoanhydride in the Series of Chlorophyll a Derivatives, Its Stability and Photoinduced Cytotoxicity
Source: Molecules. 2021 Dec 1;26(23):7298. doi: 10.3390/molecules26237298 (PMC8659179; doi:10.3390/molecules26237298)
Supplement: Supplementary file 1 [file molecules-26-07298-s001.zip › molecules-1465176-supplementary.pdf]

Supporting information

The First Selenoanhydride in the Series of Chlorophyll a Derivatives, Its Stability and Photoinduced Cytotoxicity

Viktor Pogorilyy, Anna Plyutinskaya, Nikita Suvorov, Ekaterina Diachkova, Yuriy Vasil'ev, Andrei Pankratov, Andrey Mironov and Mikhail Grin

CONTENTS

Instrumental procedures of analysis 2

Photoinduced and dark activity of compounds 1, 3, 4, and purpurinimide 3

Instrumental procedures of analysis

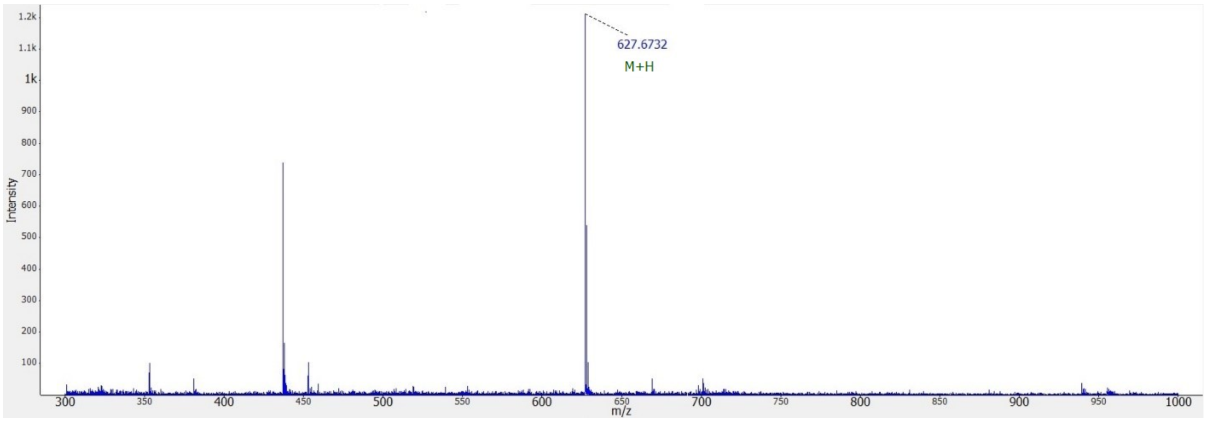

Figure S1. Mass spectrum of compound **4**.

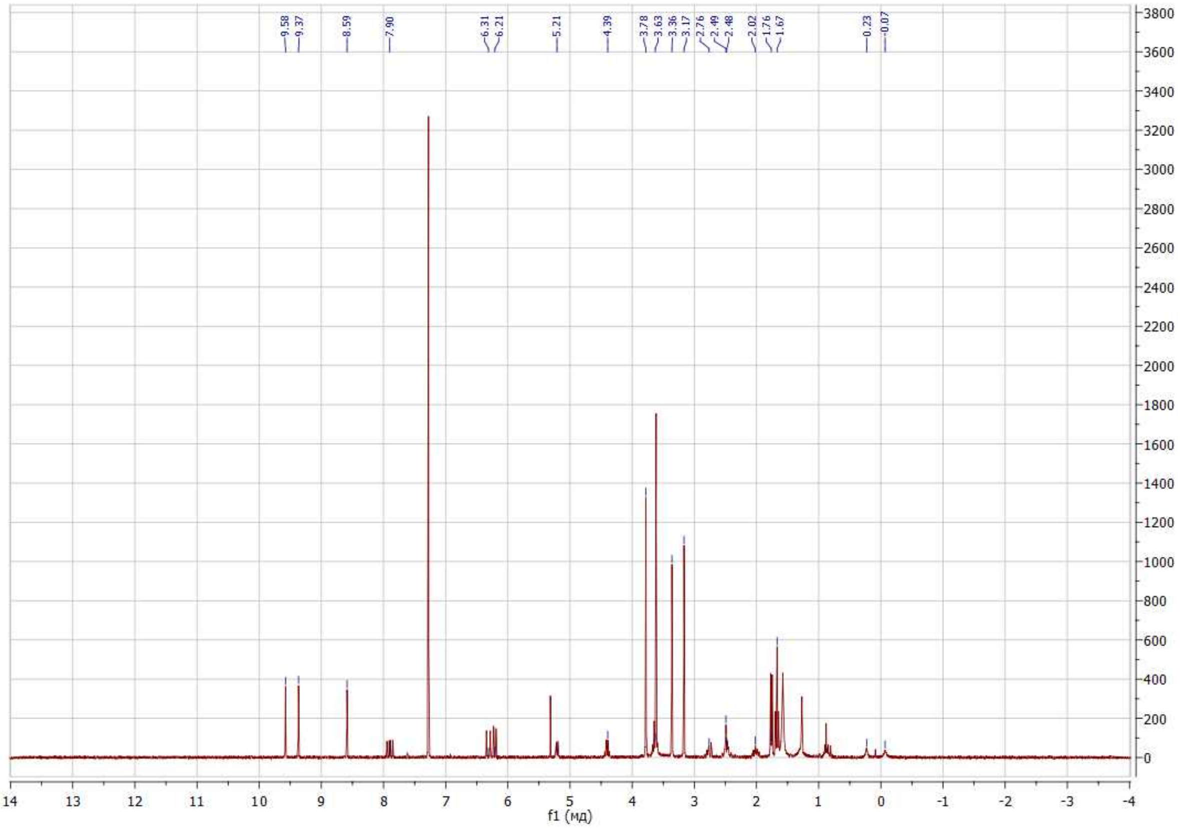

Figure S2. ^{1}H NMR spectrum of compound **4**.

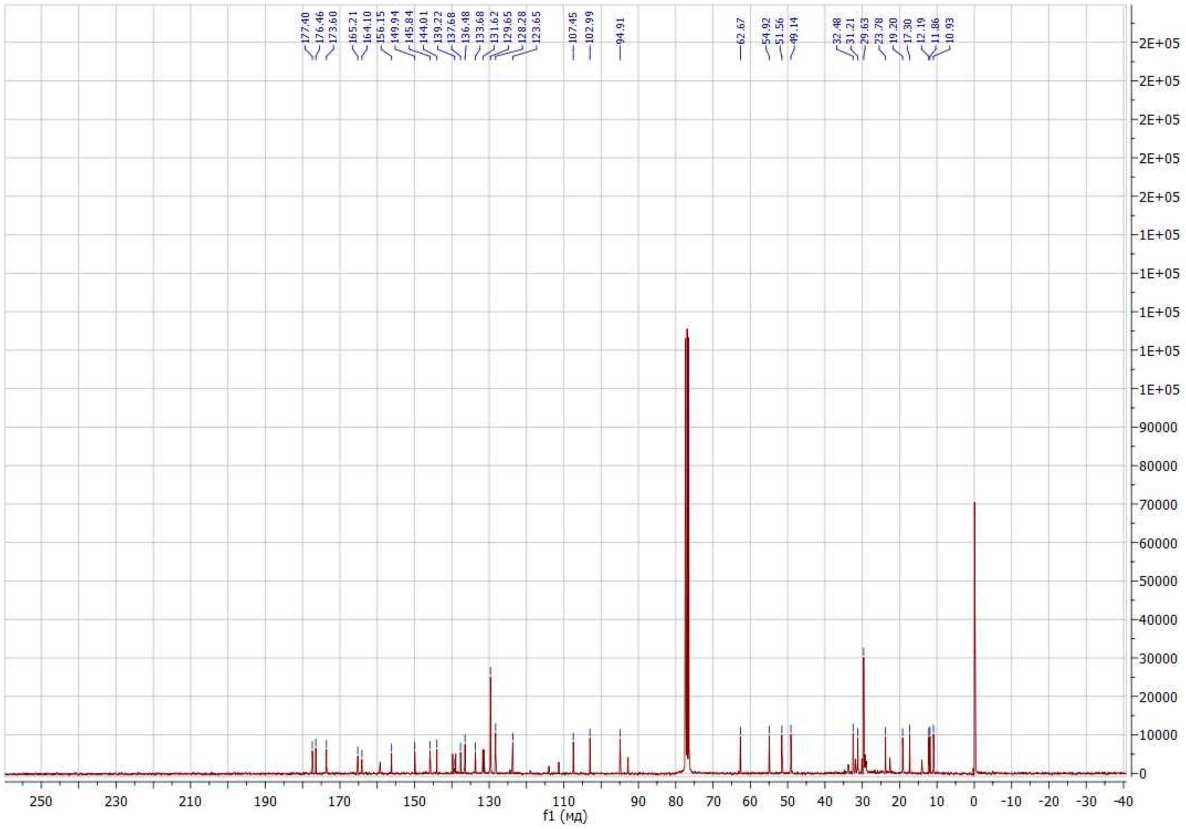

Figure S3. ^{13}C NMR spectrum of compound **4**.

Photoinduced and dark activity of compounds 1, 3, 4, and purpurinimide

Table S1. Photoinduced activity of compounds **1**, **3**, **4**, and purpurinimide.

**Compounds**	**Photo-induced activity** IC$_{50}$ value with light exposure, μg/ml	**Cytotoxic activity** IC$_{50}$ value without light exposure, μg/ml
**1**	0.43±0.04	8.5±1.1
purpurinimide	0.44±0.01	9.5±1.6
**3**	0.14±0.01	1.6±0.2
**4**	0.21±0.06	2.4±0.2
